# *N*-(2,6-Dichloro­phen­yl)succinamic acid

**DOI:** 10.1107/S1600536811024949

**Published:** 2011-07-02

**Authors:** B. S. Saraswathi, Sabine Foro, B. Thimme Gowda

**Affiliations:** a Department of Chemistry, Mangalore University, Mangalagangotri 574 199, Mangalore, India; b Institute of Materials Science, Darmstadt University of Technology, Petersenstrasse 23, D-64287 Darmstadt, Germany

## Abstract

In the crystal of the title compound, C_10_H_9_Cl_2_NO_3_, the conformations of the amide O atom and the carbonyl O atom of the acid segment are *anti* to each other and to the H atoms on the adjacent –CH_2_ groups. The C=O and O—H bonds of the acid group are *syn* to one another. In the crystal, mol­ecules are packed into infinite chains through inter­molecular O—H⋯O and N—H⋯O hydrogen bonds.

## Related literature

For our studies of the effect of substituents on the structures and other aspects of *N*-(ar­yl)-amides, see: Bhat & Gowda (2000 ▶); Gowda *et al.* (2000 ▶, 2009**a* ▶,b* ▶). For modes of inter­linking carb­oxy­lic acids by hydrogen bonds, see: Leiserowitz (1976 ▶). For packing of mol­ecules involving dimeric hydrogen-bonding associations of each carboxyl group with a centrosymmetrically related neighbor, see: Jagannathan *et al.* (1994 ▶).
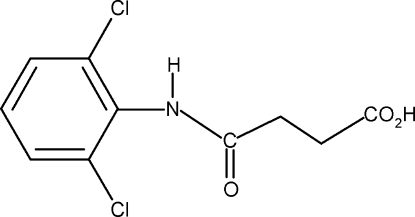


## Experimental

### 

#### Crystal data

C_10_H_9_Cl_2_NO_3_
*M* *_r_* = 262.08
Monoclinic, 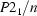

*a* = 4.713 (1) Å
*b* = 11.963 (3) Å
*c* = 20.687 (4) Å
β = 94.64 (2)°
*V* = 1162.5 (4) Å^3^
*Z* = 4
Mo *K*α radiation
μ = 0.55 mm^−1^
*T* = 293 K
0.48 × 0.06 × 0.04 mm

#### Data collection

Oxford Diffraction Xcalibur diffractometer with a Sapphire CCD detector
Absorption correction: multi-scan (*CrysAlis RED*; Oxford Diffraction, 2009 ▶) *T*_min_ = 0.779, *T*_max_ = 0.978
3912 measured reflections
1965 independent reflections
1189 reflections with *I* > 2σ(*I*)
*R*_int_ = 0.052

#### Refinement

*R*[*F* ^2^ > 2σ(*F* ^2^)] = 0.119
*wR*(*F* ^2^) = 0.222
*S* = 1.34
1965 reflections
145 parameters
18 restraints
H-atom parameters constrained
Δρ_max_ = 0.57 e Å^−3^
Δρ_min_ = −0.55 e Å^−3^

### 

Data collection: *CrysAlis CCD* (Oxford Diffraction, 2009 ▶); cell refinement: *CrysAlis RED* (Oxford Diffraction, 2009 ▶); data reduction: *CrysAlis RED*; program(s) used to solve structure: *SHELXS97* (Sheldrick, 2008 ▶); program(s) used to refine structure: *SHELXL97* (Sheldrick, 2008 ▶); molecular graphics: *PLATON* (Spek, 2009 ▶); software used to prepare material for publication: *SHELXL97*.

## Supplementary Material

Crystal structure: contains datablock(s) I, global. DOI: 10.1107/S1600536811024949/sj5173sup1.cif

Structure factors: contains datablock(s) I. DOI: 10.1107/S1600536811024949/sj5173Isup2.hkl

Supplementary material file. DOI: 10.1107/S1600536811024949/sj5173Isup3.cml

Additional supplementary materials:  crystallographic information; 3D view; checkCIF report

## Figures and Tables

**Table 1:** Hydrogen-bond geometry (Å, °)

*D*—H⋯*A*	*D*—H	H⋯*A*	*D*⋯*A*	*D*—H⋯*A*
N1—H1*N*⋯O1^i^	0.86	2.06	2.875 (9)	159
O2—H2*O*⋯O3^ii^	0.82	1.89	2.678 (11)	162

Symmetry codes: (i) 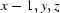; (ii) 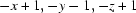.

**Fig. 1.**
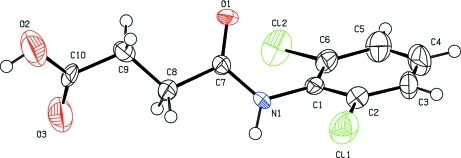
Molecular structure of the title compound, showing the atom labelling scheme and displacement ellipsoids are drawn at the 50% probability level.

**Fig. 2.**
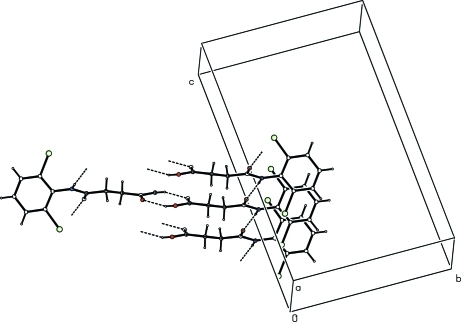
Molecular packing of the title compound with hydrogen bonding shown as dashed lines.

## supplementary crystallographic information

### Comment

The amide moiety is an important constituent of many biologically important compounds. As part of our studies into the substituent effects on the structures and other aspects of this class of compounds (Bhat & Gowda, 2000; Gowda *et al.*, 2000, 2009**a*,b*), in the present work, the crystal structure of *N*-(2,6-dichlorophenyl)-succinamic acid (I) has been determined (Fig. 1). The conformation of the amide O atom and the carbonyl O atom of the acid segment are *anti* to each other and are also *anti* to the H atoms attached to the adjacent C atoms (Fig.1). Further, C=O and O—H bonds of the acid group are *syn* to each other, similar to that observed in *N*-(2-chlorophenyl)-succinamic acid (Gowda *et al.*, 2009*a*) and *N*-(2,6-dimethylphenyl)-succinamic acid (Gowda *et al.*, 2009*b*).

In the structure, the intermolecular O—H···O and N—H···O hydrogen bonds pack the molecules into infinite chains (Table 1, Fig.2).

The modes of interlinking carboxylic acids by hydrogen bonds is described elsewhere (Leiserowitz, 1976). The packing of molecules involving dimeric hydrogen bonded association of each carboxyl group with a centrosymmetrically related neighbor has also been observed (Jagannathan *et al.*, 1994).

### Experimental

A solution of succinic anhydride (0.01 mole) in toluene (25 ml) was treated dropwise with the solution of 2,6-dichloroaniline (0.01 mole) also in toluene (20 ml) with constant stirring. The resulting mixture was stirred for about one hour and set aside for an additional hour at room temperature for completion of the reaction. The mixture was then treated with dilute hydrochloric acid to remove the unreacted 2,6-dichloroaniline. The resultant title compound was filtered under suction and washed thoroughly with water to remove the unreacted succinic anhydride and succinic acid. It was recrystallized to constant melting point from ethanol. The purity of the compound was checked and characterized by its infrared and NMR spectra.

Colorless needle like single crystals used in X-ray diffraction studies were grown in ethanolic solution by slow evaporation at room temperature.

### Refinement

The H atoms were positioned with idealized geometry using a riding model with the aromatic C—H = 0.93 Å, methylene C—H = 0.97 Å, N—H = 0.86 Å and O—H = 0.82 Å, and were refined with isotropic displacement parameters (set to 1.2 times of the *U*_eq_ of the parent atom).

The crystals available for X-ray analysis were of rather poor quality and weak scatterers at high theta value with very low intensity, resulting in relatively high *R* values. The crystal has 37.1% weak reflections.

### Crystal data

**Table:**

C_10_H_9_Cl_2_NO_3_	*F*(000) = 536
*M**_r_* = 262.08	*D*_x_ = 1.497 Mg m^−^^3^
Monoclinic, *P*2_1_/*n*	Mo *K*α radiation, λ = 0.71073 Å
Hall symbol: -P 2yn	Cell parameters from 777 reflections
*a* = 4.713 (1) Å	θ = 3.0–27.7°
*b* = 11.963 (3) Å	µ = 0.55 mm^−^^1^
*c* = 20.687 (4) Å	*T* = 293 K
β = 94.64 (2)°	Needle, colourless
*V* = 1162.5 (4) Å^3^	0.48 × 0.06 × 0.04 mm
*Z* = 4	

### Data collection

**Table:**

Oxford Diffraction Xcalibur diffractometer with a Sapphire CCD detector	1965 independent reflections
Radiation source: fine-focus sealed tube	1189 reflections with *I* > 2σ(*I*)
graphite	*R*_int_ = 0.052
Rotation method data acquisition using ω scans	θ_max_ = 25.2°, θ_min_ = 3.4°
Absorption correction: multi-scan (*CrysAlis RED*; Oxford Diffraction, 2009)	*h* = −5→5
*T*_min_ = 0.779, *T*_max_ = 0.978	*k* = −12→14
3912 measured reflections	*l* = −16→24

### Refinement

**Table:**

Refinement on *F*^2^	Primary atom site location: structure-invariant direct methods
Least-squares matrix: full	Secondary atom site location: difference Fourier map
*R*[*F*^2^ > 2σ(*F*^2^)] = 0.119	Hydrogen site location: inferred from neighbouring sites
*wR*(*F*^2^) = 0.222	H-atom parameters constrained
*S* = 1.34	*w* = 1/[σ^2^(*F*_o_^2^) + (0.*P*)^2^ + 8.7677*P*] where *P* = (*F*_o_^2^ + 2*F*_c_^2^)/3
1965 reflections	(Δ/σ)_max_ < 0.001
145 parameters	Δρ_max_ = 0.57 e Å^−^^3^
18 restraints	Δρ_min_ = −0.55 e Å^−^^3^

### Special details

**Table:**

Geometry. All e.s.d.'s (except the e.s.d. in the dihedral angle between two l.s. planes) are estimated using the full covariance matrix. The cell e.s.d.'s are taken into account individually in the estimation of e.s.d.'s in distances, angles and torsion angles; correlations between e.s.d.'s in cell parameters are only used when they are defined by crystal symmetry. An approximate (isotropic) treatment of cell e.s.d.'s is used for estimating e.s.d.'s involving l.s. planes.
Refinement. Refinement of *F*^2^ against ALL reflections. The weighted *R*-factor *wR* and goodness of fit *S* are based on *F*^2^, conventional *R*-factors *R* are based on *F*, with *F* set to zero for negative *F*^2^. The threshold expression of *F*^2^ > σ(*F*^2^) is used only for calculating *R*-factors(gt) *etc*. and is not relevant to the choice of reflections for refinement. *R*-factors based on *F*^2^ are statistically about twice as large as those based on *F*, and *R*- factors based on ALL data will be even larger.

### Fractional atomic coordinates and isotropic or equivalent isotropic displacement parameters (Å^2^)

**Table:**

	*x*	*y*	*z*	*U*_iso_*/*U*_eq_	
C1	0.5925 (18)	0.1082 (7)	0.3449 (4)	0.028 (2)	
C2	0.4791 (19)	0.1566 (8)	0.2870 (5)	0.039 (2)	
C3	0.554 (2)	0.2620 (8)	0.2688 (5)	0.049 (3)	
H3	0.4712	0.2925	0.2304	0.059*	
C4	0.749 (3)	0.3227 (9)	0.3068 (6)	0.063 (4)	
H4	0.8010	0.3938	0.2938	0.076*	
C5	0.871 (2)	0.2786 (9)	0.3643 (6)	0.058 (3)	
H5	1.0064	0.3186	0.3899	0.069*	
C6	0.785 (2)	0.1730 (8)	0.3832 (5)	0.041 (3)	
C7	0.6868 (18)	−0.0859 (7)	0.3757 (4)	0.027 (2)	
C8	0.5518 (18)	−0.1883 (8)	0.4030 (5)	0.039 (2)	
H8A	0.3759	−0.2043	0.3771	0.047*	
H8B	0.5035	−0.1716	0.4467	0.047*	
C9	0.7363 (19)	−0.2911 (7)	0.4051 (4)	0.034 (2)	
H9A	0.7487	−0.3182	0.3613	0.041*	
H9B	0.9269	−0.2711	0.4225	0.041*	
C10	0.627 (2)	−0.3827 (8)	0.4453 (5)	0.044 (3)	
N1	0.5076 (14)	0.0022 (6)	0.3650 (3)	0.0312 (18)	
H1N	0.3308	−0.0076	0.3709	0.037*	
O1	0.9365 (12)	−0.0820 (5)	0.3658 (3)	0.0348 (16)	
O2	0.738 (2)	−0.4776 (7)	0.4371 (4)	0.095 (3)	
H2O	0.6475	−0.5255	0.4551	0.114*	
O3	0.460 (2)	−0.3676 (7)	0.4844 (4)	0.089 (3)	
Cl1	0.2395 (6)	0.0781 (3)	0.23715 (14)	0.0625 (9)	
Cl2	0.9252 (7)	0.1234 (3)	0.45814 (13)	0.0621 (9)	

### Atomic displacement parameters (Å^2^)

**Table:**

	*U*^11^	*U*^22^	*U*^33^	*U*^12^	*U*^13^	*U*^23^
C1	0.024 (5)	0.017 (5)	0.046 (6)	0.003 (4)	0.012 (4)	−0.001 (4)
C2	0.033 (6)	0.038 (6)	0.047 (6)	0.006 (5)	0.007 (5)	0.009 (5)
C3	0.061 (7)	0.032 (6)	0.059 (7)	0.012 (6)	0.026 (6)	0.020 (6)
C4	0.074 (9)	0.024 (6)	0.096 (10)	−0.002 (6)	0.030 (8)	0.006 (7)
C5	0.062 (8)	0.028 (6)	0.083 (9)	−0.003 (6)	0.009 (7)	−0.003 (6)
C6	0.045 (6)	0.029 (6)	0.051 (7)	0.012 (5)	0.017 (5)	0.002 (5)
C7	0.023 (5)	0.023 (5)	0.035 (5)	−0.003 (4)	0.000 (4)	0.004 (4)
C8	0.025 (5)	0.031 (6)	0.059 (7)	0.001 (4)	0.000 (5)	0.016 (5)
C9	0.032 (5)	0.030 (5)	0.042 (6)	−0.003 (4)	0.013 (4)	0.002 (4)
C10	0.048 (6)	0.022 (5)	0.065 (7)	0.021 (5)	0.025 (5)	0.015 (5)
N1	0.019 (4)	0.027 (4)	0.049 (5)	−0.003 (3)	0.010 (3)	0.014 (3)
O1	0.018 (3)	0.028 (4)	0.059 (4)	−0.001 (3)	0.011 (3)	0.005 (3)
O2	0.119 (7)	0.057 (5)	0.120 (6)	0.012 (5)	0.075 (5)	0.031 (5)
O3	0.112 (6)	0.048 (5)	0.116 (6)	0.021 (5)	0.068 (5)	0.028 (5)
Cl1	0.0596 (18)	0.063 (2)	0.0619 (19)	0.0017 (16)	−0.0145 (14)	0.0092 (15)
Cl2	0.080 (2)	0.0543 (18)	0.0492 (17)	0.0067 (16)	−0.0129 (15)	−0.0084 (15)

### Geometric parameters (Å, °)

**Table:**

C1—C6	1.392 (13)	C7—N1	1.358 (10)
C1—C2	1.396 (12)	C7—C8	1.511 (12)
C1—N1	1.402 (10)	C8—C9	1.505 (12)
C2—C3	1.370 (13)	C8—H8A	0.9700
C2—Cl1	1.741 (10)	C8—H8B	0.9700
C3—C4	1.370 (15)	C9—C10	1.491 (12)
C3—H3	0.9300	C9—H9A	0.9700
C4—C5	1.382 (15)	C9—H9B	0.9700
C4—H4	0.9300	C10—O3	1.190 (11)
C5—C6	1.391 (14)	C10—O2	1.266 (11)
C5—H5	0.9300	N1—H1N	0.8600
C6—Cl2	1.739 (10)	O2—H2O	0.8200
C7—O1	1.212 (9)		
			
C6—C1—C2	116.4 (8)	N1—C7—C8	114.5 (7)
C6—C1—N1	121.6 (8)	C7—C8—C9	114.4 (7)
C2—C1—N1	122.0 (8)	C7—C8—H8A	108.7
C3—C2—C1	121.9 (10)	C9—C8—H8A	108.7
C3—C2—Cl1	120.1 (8)	C7—C8—H8B	108.7
C1—C2—Cl1	118.0 (7)	C9—C8—H8B	108.7
C2—C3—C4	120.4 (11)	H8A—C8—H8B	107.6
C2—C3—H3	119.8	C10—C9—C8	113.1 (7)
C4—C3—H3	119.8	C10—C9—H9A	109.0
C3—C4—C5	120.2 (11)	C8—C9—H9A	109.0
C3—C4—H4	119.9	C10—C9—H9B	109.0
C5—C4—H4	119.9	C8—C9—H9B	109.0
C4—C5—C6	118.7 (11)	H9A—C9—H9B	107.8
C4—C5—H5	120.7	O3—C10—O2	121.9 (9)
C6—C5—H5	120.7	O3—C10—C9	123.1 (9)
C5—C6—C1	122.4 (10)	O2—C10—C9	114.9 (8)
C5—C6—Cl2	117.6 (9)	C7—N1—C1	124.2 (7)
C1—C6—Cl2	120.0 (7)	C7—N1—H1N	117.9
O1—C7—N1	122.9 (8)	C1—N1—H1N	117.9
O1—C7—C8	122.6 (8)	C10—O2—H2O	109.5
			
C6—C1—C2—C3	0.2 (13)	C2—C1—C6—Cl2	176.8 (7)
N1—C1—C2—C3	177.3 (8)	N1—C1—C6—Cl2	−0.4 (12)
C6—C1—C2—Cl1	179.4 (7)	O1—C7—C8—C9	−11.0 (13)
N1—C1—C2—Cl1	−3.4 (11)	N1—C7—C8—C9	170.9 (8)
C1—C2—C3—C4	1.5 (15)	C7—C8—C9—C10	167.1 (9)
Cl1—C2—C3—C4	−177.8 (8)	C8—C9—C10—O3	−18.2 (16)
C2—C3—C4—C5	−0.9 (17)	C8—C9—C10—O2	165.2 (10)
C3—C4—C5—C6	−1.3 (17)	O1—C7—N1—C1	−3.5 (14)
C4—C5—C6—C1	3.1 (15)	C8—C7—N1—C1	174.5 (8)
C4—C5—C6—Cl2	−176.2 (8)	C6—C1—N1—C7	−62.7 (12)
C2—C1—C6—C5	−2.5 (13)	C2—C1—N1—C7	120.3 (10)
N1—C1—C6—C5	−179.6 (9)		

### Hydrogen-bond geometry (Å, °)

**Table:**

*D*—H···*A*	*D*—H	H···*A*	*D*···*A*	*D*—H···*A*
N1—H1N···O1^i^	0.86	2.06	2.875 (9)	159
O2—H2O···O3^ii^	0.82	1.89	2.678 (11)	162

Symmetry codes: (i) *x*−1, *y*, *z*; (ii) −*x*+1, −*y*−1, −*z*+1.
